# Commentary: “Large-scale psychological differences within China explained by rice vs. wheat agriculture”

**DOI:** 10.3389/fpsyg.2015.00489

**Published:** 2015-04-22

**Authors:** Shihu Hu, Zhiguo Yuan

**Affiliations:** Advanced Water Management Centre, Faculty of Engineering, Architecture and Information Technology, The University of QueenslandBrisbane, QLD, Australia

**Keywords:** China, rice vs. wheat, rice vs. non-rice, individualism, collectivism

Talhelm et al. (2014) reported an interesting investigation on the psychological differences between Southern and Northern Han Chinese populations in China. By interviewing 1162 undergraduate students from different regions, the authors showed that the Chinese from the southern “rice-growing area” are more holistic-thinking and interdependent, while the Chinese from the northern “wheat-growing area” are more analytic-thinking and independent. These psychological differences can be explained by the differences between rice and wheat farming, not the modernization theory. The authors suggested that this “rice vs. wheat” theory can partially explain the different thought styles between Eastern and Western people, and the persistent interdependence of wealthy East Asia.

We think that some relevant detailed data have been overlooked in their analysis, which has significant implications for the paper's conclusion and subsequent discussions. When comparing wheat with rice statistics, the authors grouped the corn and soybeans farming area with the wheat area (Talhelm et al., 2014). This simplified method may be valid when wheat dominates over corn and soybeans, however, this does not uniformly occur in Northern China.

We downloaded the farm crops area data from the website of the State Statistical Bureau of the People's Republic of China (1996), the same source of data as used by Talhelm et al. (2014), and compared the four most widely planted crops in China in 1996, namely rice, wheat, corn, and soybeans. While the authors grouped together wheat, corn, and soybeans to compare with rice during the analysis, we compared each crop respectively against rice.

We found that, (1) In Northern China, corn and wheat are equally important crops, and in many provinces corn is more widely planted than wheat; (2) Similar to wheat, the percentage of corn farm is also negatively correlated, r_(27)_ = −0.60, *P* < 0.001, to that of rice at the provincial level; (3) In several provinces that were categorized by the authors as “wheat provinces” e.g., Jilin and Liaoning provinces (in contrast to the “rice provinces”), there were actually many more rice fields than wheat (Figure 1).

**Figure 1:**
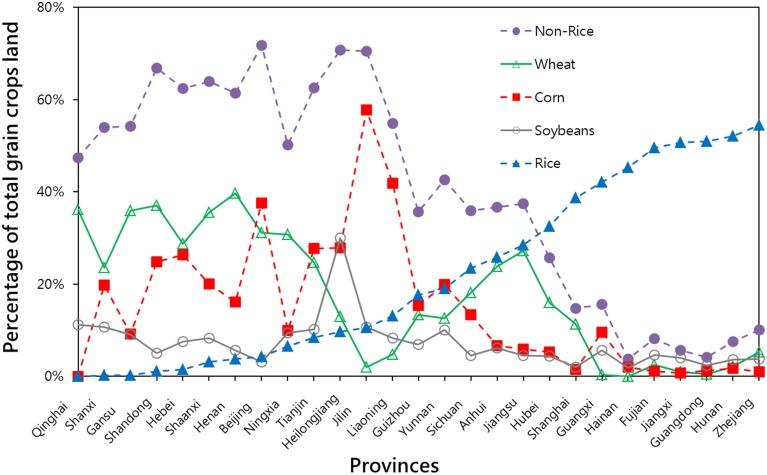
**Comparison between the percentages of planting areas of four different grain crops in China, 1996**. Non-Rice was defined as the sum of corn, wheat and soybeans. Other types of grain crops were planted (data not available), therefore, the sum of all four named crops is lower than 100%.

These results suggest that there would be an equal chance for corn farming to affect population psychology in Northern China. The term “rice vs. wheat agriculture” coined by Talhelm et al. (2014) cannot accurately reflect their findings. From their data, it seems to be more appropriate to attribute the psychological differences to “rice vs. non-rice agriculture”.

The detailed data which were overlooked not only affect the conclusion of Talhelm et al. (2014), but also the implications. With the “rice vs. wheat agriculture” theory, the paper implied that the different thought styles between East and West may be partially explained by respectively different agricultures. However, the amendment of “rice vs. wheat agriculture” to “rice vs. non-rice agriculture” in the hypothesis would lead to different implications.

Thus the theory would then predict that there should be psychological differences between rice-growing East/South East Asian and the rest of world. This prediction is inconsistent with the dominant view in psychology that Western populations show different psychological traits as compared with non-Western populations. Non-Westerns, which include groups as diverse as Arabs, East Asians, Russians, and farmers in Africa and South America, have been shown to rely more on holistic reasoning and have more interdependent views of self than Westerners (Henrich et al., 2010). Data from the United States Department of Agriculture Foreign Agricultural Service (2014) show that for many of these non-Westerners, e.g., people from the South and Middle America, and Middle East regions, non-rice crops such as corn or wheat are dominant over rice. According to the “rice vs. non-rice agriculture” theory, they are expected to be more similar to Westerners. However, it has been shown that these people demonstrate the same level of, or increased holistic processing and collectivism as compared to Chinese people (Allik and Realo, 2004; Henrich et al., 2010).

This paper is well publicized, and has led to much discussion within both the general public and the scientific community, including a critical commentary by Ruan et al. (2014). Many have used the “rice vs. wheat agriculture” theory to explain the psychological differences between East and West, and why Europe, rather than China, became the center of industrial revolution and innovation (Biello, 2014; Henrich, 2014; MacKenzie, 2014). If the more appropriate “rice vs. non-rice agriculture” theory was proposed, and the potential role of corn in affecting Chinese psychology was adequately presented, the readers would have discussed the results in a broader context, and exercised more caution in extrapolating the results to explain the differences between East and West.

Furthermore, the psychological differences observed by Talhelm et al. (2014) cannot be because of growing certain crops has an inherent influence on psychology. Instead, the real underlying driving force for the psychological differences is likely the different labor intensity and different levels of collaboration required when farming different crops (Vandello and Cohen, 1999). “Rice vs. wheat” or even “rice vs. non-rice” are superficial comparisons, and could be misleading since the productions costs of rice/wheat/corn in different parts of the world likely vary depending on the local conditions. The analysis of current and future data should focus on the impact of different production costs of different agricultures, instead of these agricultures themselves, on individualism vs. collectivism.

Indeed, while the cost of wheat production is much less than that of rice, the cost of corn production is between the two or closer to that of rice in modern China (Garnaut et al., 2013). This suggests that wheat and corn should be separately analyzed in contrast to rice, when evaluating the influence of their production on human psychology.

Talhelm et al. (2014) provides a new angle for unearthing the driving force behind the psychological differences of respective populations. However, it appears that the conclusion and discussion are based on an overly simplified interpretation of the data. We hope our discussion helps to establish a more accurate boundary for this new theory, and help researchers to accurately define research questions and design their experiments accordingly in future studies.

## Conflict of interest statement

The authors declare that the research was conducted in the absence of any commercial or financial relationships that could be construed as a potential conflict of interest.
